# Single nucleotide polymorphism-specific regulation of matrix metalloproteinase-9 by multiple miRNAs targeting the coding exon

**DOI:** 10.1093/nar/gku197

**Published:** 2014-03-13

**Authors:** Tyler Duellman, Christopher Warren, Jay Yang

**Affiliations:** 1 Molecular and Cellular Pharmacology Graduate Program, University of Wisconsin, SMI 301, 1300 University Ave., Madison, WI 53706, USA; 2 Proteovista LLC, Madison, WI 53719, USA; 3 Department of Anesthesiology, University of Wisconsin, Madison, WI 53706, USA

## Abstract

Microribonucleic acids (miRNAs) work with exquisite specificity and are able to distinguish a target from a non-target based on a single nucleotide mismatch in the core nucleotide domain. We questioned whether miRNA regulation of gene expression could occur in a single nucleotide polymorphism (SNP)-specific manner, manifesting as a post-transcriptional control of expression of genetic polymorphisms. In our recent study of the functional consequences of matrix metalloproteinase (MMP)-9 SNPs, we discovered that expression of a coding exon SNP in the pro-domain of the protein resulted in a profound decrease in the secreted protein. This missense SNP results in the N38S amino acid change and a loss of an N-glycosylation site. A systematic study demonstrated that the loss of secreted protein was due not to the loss of an N-glycosylation site, but rather an SNP-specific targeting by miR-671-3p and miR-657. Bioinformatics analysis identified 41 SNP-specific miRNA targeting MMP-9 SNPs, mostly in the coding exon and an extension of the analysis to chromosome 20, where the MMP-9 gene is located, suggesting that SNP-specific miRNAs targeting the coding exon are prevalent. This selective post-transcriptional regulation of a target messenger RNA harboring genetic polymorphisms by miRNAs offers an SNP-dependent post-transcriptional regulatory mechanism, allowing for polymorphic-specific differential gene regulation.

## INTRODUCTION

The human genome encodes about 20 000 protein coding genes, which occupy just 1.5% of the human genome (1). The key contribution of the non-coding genome in the regulation of gene expression is now well established. Microribonucleic acids (miRNAs) are one class of non-coding RNAs that target messenger RNA (mRNA) through core nucleotide domain pairing (2), regulating mRNA stability and/or translational efficiency with many identified targets relevant for normal development or disease conditions (3–6). miRNAs work with exquisite specificity: they distinguish a target from a non-target based on a single nucleotide mismatch in the core nucleotide domain (7) with a consequent reduction in the protein output (8,9) upon translational inhibition and mRNA destabilization (10). This is likely mediated by deadenylation and decapping of the targeted mRNA (11). A genome-wide computational analysis focusing on the 3’ untranslated region (UTR) of genes suggested the presence of a large number of single nucleotide polymorphisms (SNPs) in human miRNA targets (12), implying a potential SNP-dependent effect of miRNA regulation. An SNP-dependent creation of ‘illegitimate miRNA binding site’ in the 3’UTR of the myostatin gene affecting muscularity in sheep has been reported (13). The importance of polymorphisms in miRNA target sites (poly-miRTS) in diseases has been suggested (14) and recent reports, for example, suggest its potential role in cancer (15,16). Both bioinformatics and experimental analyses have now been extended to confirm miRNA targets in the coding exons as well (17–19) but with limited information available on the impact of coding exon SNPs on miRNA regulation.

Matrix metalloproteinases (MMPs), a family of Zn^++^-dependent endopeptidases, play a key role in extracellular matrix (ECM) remodeling, especially in elastin and collagen turnover (20). Increased levels of the gelatinase subset (MMP-2 and -9) of this protease family are found in developing aortic aneurysms (21,22). Association between specific genotypes of these MMPs and incidence of unstable arterial plaque rupture leading to acute myocardial infarction have also been reported (23). MMP-9 activity is important not only for cardiovascular diseases but also for many diseases where ECM may play a pathogenic role, including cancer metastasis, lumbar disk herniation, chronic obstructive pulmonary disease, autoimmune diseases, vascularization and skeletal growth (24–26). MMP-9 is an inducible enzyme mostly regulated at the transcriptional level. However, like other members of the MMP family, the protein is secreted as a pro-enzyme, activated when the N-terminus pro-domain unfolds and is cleaved by activator proteases exposing the catalytic domain (27), adding a significant post-transcriptional element to its regulation. In addition to these traditional mechanisms of MMP-9 regulation, recent reports indicate a level of epigenetic and miRNA regulation (28), adding further complexity to the regulation of this key enzyme essential to ECM remodeling. A deeper understanding of MMP-9 regulation is critical to better defining the role of this multifunctional protein in normal biology and pathoetiology.

In our recent study aimed to better understand the functional consequences of MMP-9 SNPs, we characterized a coding exon SNP in the pro-domain of the protein (N38S, rs41427445) that resulted in a profound decrease in the secreted protein (29). We questioned whether miRNA regulation of MMP-9 expression could occur in an SNP-specific manner, manifesting as a post-transcriptional control of expression of genetic polymorphisms in the protein coding exons. Our results demonstrate an SNP-specific regulation of MMP-9 through miRNA targeting the coding region of the gene. Bioinformatics analysis revealed SNP-specific regulation of MMP-9 by additional miRNA targeting other SNPs, including synonymous SNPs, with no change in the coded amino acid. This discovery reveals a cellular mechanism whereby expression of a specific MMP-9 mRNA is affected by a highly selective miRNA interaction with the SNP-mRNA, most likely playing an important role in the biology of MMP-9. Extension of the analysis to other genes located on chromosome (Chr) 20 indicates that this novel SNP-specific miRNA regulation of target mRNA is likely to be a prevalent mechanism that affects many genes.

## MATERIALS AND METHODS

### Creation of MMP-9 clones with the desired SNPs and enzymatic activity assay

Starting with the wild-type human MMP-9 complementary deoxyribonucleic acid (cDNA; Accession BC006093, Image Clone MGC: 12688), we created the less prevalent exon genotypes by introducing the desired nucleotide switch by overlap polymerase chain reaction (PCR), using the primers listed in Supplementary Table S1. All final constructs were sequenced to confirm the presence of the desired mutagenesis and the lack of unintended base changes due to PCR.

The cDNAs containing the exon SNPs were subcloned into an expression vector (pCI/neo; Promega, Madison, WI, USA) and the protein expressed through transient transfection of the human embryonic kidney (HEK) 293 cells (ATCC, Manassas, VA, USA) using Lipofectamine 2000 (Life Technologies, Grand Island, NY, USA). HEK293 cells were grown in Dulbecco's Modified Eagle's medium (Mediatech Inc., Manassas, VA, USA) with 4.5-g/l glucose, L-glutamine, sodium and pyruvate supplemented with 10% heat inactivated fetal bovine serum (Mediatech Inc.), 100-U/ml penicillin and 0.1-mg/ml streptomycin. The culture supernatant was harvested 24 h after transfection and MMP-9 activated by *in vitro* treatment with 4-aminophenylmercuric acetate, activating the pro-enzyme by unfolding the protein. The MMP-9 enzymatic activity was assayed by gelatin zymography and quantified with gel densitometry. Briefly, 0.75-mm sodium dodecyl sulphate-polyacrylamide gel electrophoresis (SDS-PAGE) gels were prepared with the incorporation of gelatin (1 mg/ml) before casting. Denatured but non-reduced samples and standards were run at constant voltage. Gels were allowed to renature by four washes in an enzyme renaturing buffer containing 2.5% v/v Triton X-100 for 1 h. Gels were then incubated at 37°C for an extended period of time, followed by staining with Coomassie Brilliant Blue 250-R. After destaining, zones of enzyme activity showed up as clear bands and were quantified by densitometric analysis of the inverted image. Preliminary studies confirmed that the amount of transfected plasmids (0.5 μg/well of a 6-well plate) and gel loading all fell within a linear range of densitometry (29), and in general gave a more reproducible result compared to activity assessment with a kinetic measurement of the cleavage of a fluorogenic MMP-9 substrate (Enzo-Biomol, Farmingdale, NY, USA) (data not shown). A western blot of culture supernatant with the anti-MMP-9 (1:1000 in 1% milk Tris buffered saline with 0.1% Triton X-100 (TBST), Clone L51/82, NeuroMab, Davis, CA, USA) antibody followed by densitometric quantification documented the amount of secreted MMP-9 protein. Since no good loading control for the culture supernatant could be found, we empirically determined the linear range of correlation between the total cell lysate and the culture supernatant (Supplementary Figure S1). Loading up to 20 μl of the culture supernatant gave a linear signal intensity for the transfection condition used. Total secreted MMP-9 was also quantified by the hMMP-9 ELISA kit (#KHC3061, Invitrogen, Camarillo, CA, USA). ELISA was carried out according to vendor's recommendation. The linearity of the assay confirmed for MMP-9 concentrations within 0–1500 pg/ml. HEK293 supernatant was diluted (1:1000) to attain an appropriate concentration within the linear range and loaded in triplicate in a 96-well format. MMP-9 protein amount was quantified via colorimetric detection using a microplate reader. N-linked glycan residues were enzymatically removed using PNGase F (ProZyme Inc., San Leandro, CA, USA). Briefly, MMP-9 protein was denatured at 100°C for 5 min in 0.1% SDS, 50-mM β-ME, 0.75% NP-40 and 50-mM sodium phosphate. Denatured MMP-9 was then incubated with 0.015 U of PNGase F for 3 h at 37°C. Cell culture analysis of N-linked glycosylation and proteasomal degradation was inhibited with 24-h treatment of tunicamycin (1.0 μg/mL) (Santa Cruz Biotechnology Inc., Dallas, TX, USA) and MG132 (10 μM) (Tocris Bioscience, Bristol, UK), respectively.

### Determination of MMP-9 mRNA stability

HEK293 cells were plated at a density of 1×10^5^ cells per well in a 12-well plate 12 h prior to transfection. Three hundred seventy-five nanograms of wild type or N38S–MMP-9 pIRES2-EGFP (Promega) were transfected using Lipofectamine 2000 (Life Technologies) and OPTI-MEM (Life Technologies). Sixteen-hour post-transfections 10 μg/ml of actinomycin D (Sigma, St. Louis, MO, USA) in OPTI-MEM was administered to cells. mRNA was harvested from cells at 0, 4 and 8-h post-actinomycin D treatment. glyceraldehyde 3-phosphate dehydrogenase (GAPDH) was used as an endogenous control and samples were normalized to cells harvested at 0-h post-treatment to calculate % mRNA remaining. In addition to the obvious morphologic changes induced by the drug, the transcriptional inhibition by actinomycin D at the concentration used was confirmed by inhibition of expression of EGFP-pCI/neo in transfected cells.

### Luciferase stability (miRNA reporter) assay

Tandem wild type or N38S–MMP-9 sequences encompassing miRNA binding sites, scrambled, or the exact miRNA complementary binding site sequences were subcloned into an introduced BsmBI cleavage site in the 3’UTR of the firefly luciferase coding region of pGL3 vector using a common 4-mer overhang sequence (Promega). Subcloned oligonucleotide sequences are listed in Supplementary Table S1. HEK293 cells were plated at a density of 5×10^4^ cells per well in a 24-well plate 12 h prior to transfection. Cells were transfected with Lipofectamine 2000 (Life Technologies) and Opti-MEM (Life Technologies) with 100 ng of the luciferase 3’UTR reporter vector, 25-ng *Renilla* vector, pGL4.75 (Promega), and treated with or without miRNA antagomirs (miR-671-3p antagomir: 15–30 nM, miR-657 antagomir: 7.5–15 nM, (−) control antagomir: 15–30 nM, acting as a scrambled control) (mirVana, Life Technologies; reagent lot numbers listed in Supplementary Table S1). Five-hour post-transfection cells were harvested, re-plated in duplicate in a 96-well plate, and 30-h post-transfection the luciferase activity was measured using the Dual-Glo Luciferase Assay (Promega) according to the manufacturer's instructions.

### Qantitative reverse transcription-polymerase chain reaction (qRT-PCR)

Total RNA was isolated with RNeasy (Qiagen, Germantown, MD, USA) according to the manufacturer's instructions. cDNA was reverse transcribed from 1 μg of total RNA with a QuantiTect Reverse Transcription Kit (Qiagen). Genomic DNA and any potential plasmid DNA carryover were removed by the genomic DNA wipeout buffer included in this kit. Templates were amplified in a 25-μl reaction mixture containing 12.5-μl SYBR Jump Start Taq (Sigma-Aldrich), 0.25-μl ROX reference dye, 11-μl DNase-free H_2_O, 0.2 μM each of forward and reverse primers (see Supplementary Table S1) and 0.25-μl cDNA solution. Real-time PCR analysis was performed with a M×3000P (Stratagene, La Jolla, CA, USA) with the following amplification conditions: 95°C for 10 min followed by 40 cycles of 15 s at 95°C and 60 s at 60°C. PCR specificity was confirmed by Basic Local Alignment Search Tool search and melting curve analysis after the amplification. Relative expression was quantified using the ΔΔ*C*_t_ method. Human GAPDH, RNU48 (RT^2^ qPCR Primer Assay, Qiagen) or *Renilla* luciferase was used as an internal standard as appropriate for the experiment.

### miRNA detection and modulation

The expression of miRNA was quantified by qRT-PCR using a commercial miRNA detection kit (TaqMan MicroRNA Assays, Life Technologies; reagent lot numbers listed in Supplementary Figure S1). This method consists of a target-specific looped primer and a reporter oligonucleotide designed to hybridize to a 3’ extension to one of the specific primers. The fluorescent signal from the reporter oligonucleotide is normally quenched, reporting only when the quencher molecule is removed when incorporated into the double-stranded specific PCR product. miRNA mimics, antagomirs and control small synthetic RNA (mirVana, Life Technologies) specific to a given target miRNA were transfected following the manufacturer's recommended protocol. Non-specific suppression of MMP-9 protein expression by mirVana reagents was analyzed using western blot after transfection of increasing amounts of the negative control small RNA (0–120 nM). As per the vendor's recommendations, non-specific decrease in the expressed protein was detected with negative control small RNA exceeding 30 nM (data not shown). Subsequent experiments were performed using ≤30-nM total small RNA reagent. Further details can be found at the manufacturer's web link (http://tools.invitrogen.com/content/sfs/manuals/cms_042167.pdf).

### Immunocytochemistry

Twenty-four hours after HEK293 cells were transfected with the wild type or N38S–MMP-9 cDNA they were fixed in 4% paraformaldehyde (in 0.1-M phosphate buffer, pH7.4) and immuno-stained with anti-MMP-9 antibody (1:200, Clone L51/82, UC Davis/NIH NeuroMab Facility, Davis, CA, USA) in PBST with 2% normal goat serum for 1 h followed by an Alexa 594-conjugated secondary antibody for 1 h, both at room temperature.

### Bioinformatics

Most currently available miRNA search algorithms seeking miRNA interacting with poly-miRTS in an allele-dependent manner exclusively focus on the 3’UTR (30–32), which likely contributes to the limited number of reports of miRNA targeting the gene coding region. We implemented a *de novo* search algorithm based on the report by Nicoloso et al. (6). The 1919 mature human miRNA sequences were downloaded (July 2012) from miRBase. Annotations listing all coding exons, 5’UTRs and 3’UTRs for each gene (refFlat.txt), were downloaded (July 2012) from the University of California–Santa Cruz. The human genome sequence for hg19 (GRCh37.1), sequences surrounding each SNP (August 2011), and the position of each SNP (August 2011) were downloaded from the National Center for Biotechnology Information (NCBI). All genomic positions are based on the GRCh37.1 sequence. The miRanda software was downloaded (August 2010 release) (www.microrna.org/microrna/getDownloads.do) and implemented to run on a desktop PC with the miRanda parameters set to a gap opening penalty of −9, a gap extension penalty of −4 and a scaling factor of 4. Energy and Score thresholds were set as described in the text. A custom PERL script was written for all other string manipulations. A flow diagram describing the search strategy is shown in Figure 2a. Coding exon polymorphism types and amino acid positions based on allelic variations of SNPs were identified using data (b137_SNPContigLocusId.bcp) downloaded (June 2012) from NCBI.

**Figure 1.**
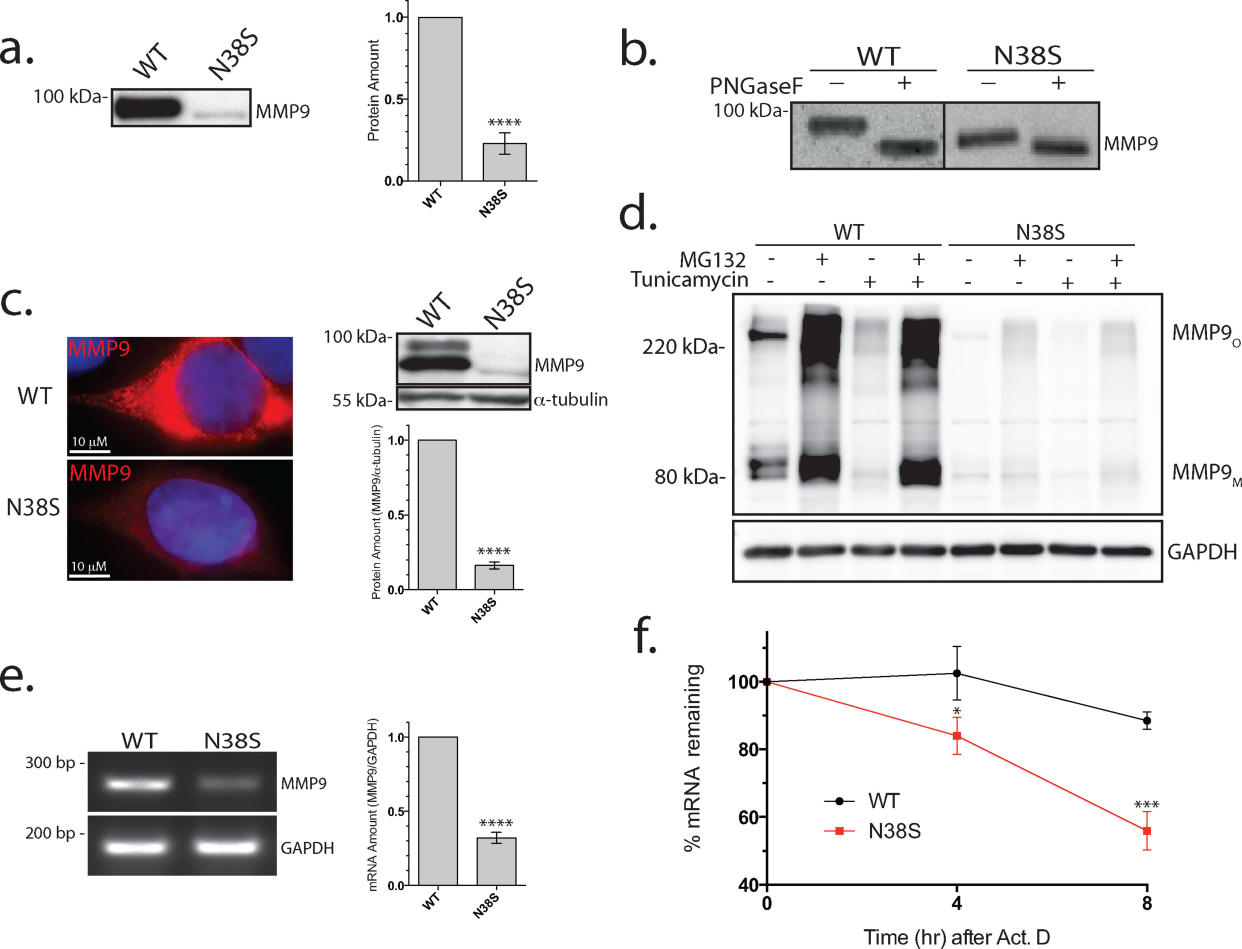
Reduction in secreted N38S–MMP-9 protein is not due solely to the loss of N-glycosylation. (**a**) Western blot analysis of culture media (20 μl) from cells transfected with wild type (WT) or N38S–MMP-9 probed with anti-MMP-9 antibody (left). Similar results obtained from five separate transfections. The MMP-9 protein amount was quantified by ELISA and averaged (mean ± SEM) from five separate transfections shown as a bar plot (right). ****P < 0.0001, t-test and *n* = 5. (**b**) Western blot of PNGase F-treated cell culture media from wild type (WT) or N38S–MMP-9-transfected cells. Note that the N38S–MMP-9 culture media were concentrated to obtain equivalent band intensity to the WT. The observed PNGase-induced mobility shift is consistent with a loss of two N-glycosylation sites for the wild type and the loss of one remaining N-glycosylation for N38S. (**c**) Anti-MMP-9 immunohistochemical staining (red) of WT and N38S–MMP-9-transfected cells. Nuclei were stained by Hoechst 33342 (blue) (left). Western blot analysis of total cell lysate probed with anti-MMP-9 or anti-α-tubulin antibody indicating reduced N38S–MMP-9 intracellular protein levels (right).****P < 0.0001, t-test and *n* = 5. (**d**) Cells expressing WT or N38S–MMP-9 were treated with or without MG132 (proteasome degradation inhibitor) (10 μM) or tunicamycin (GlcNAc phosphotransferase inhibitor; selectively inhibiting N-glycosylation) (1.0 μg/mL) both for 24 h and cell culture media ran on a non-reducing PAGE and was probed with anti-MMP-9 or anti-GAPDH antibody. Note the MMP-9 monomer (MMP-9_M_) with an apparent molecular mass near the expected ∼90 kDa and the larger oligomeric form (MMP_O_). MG132 failed to rescue the N38S–MMP-9 protein amount. Tunicamycin treatment of WT mimicked the reduced N38S protein levels, but was completely rescued by MG132. Representative of three separate experiments. (**e**) Total RNA was harvested from cells transfected with the WT or N38S–MMP-9 plasmids, and an end point RT-PCR performed with primer pairs amplifying the MMP-9 or GAPDH mRNA. Agarose gel electrophoresis demonstrated a reduction in the amplified PCR product for N38S compared to WT. ****P < 0.0001, t-test and *n* = 3. (**f**) The stability of WT or N38S–MMP-9 mRNA was examined using qRT-PCR at the indicated time points after actinomycin D treatment (10 μg/mL). The data are normalized to the respective mRNA levels at time 0 h. *P < 0.05, ***P < 0.0005, t-test and *n* = 3.

**Figure 2.**
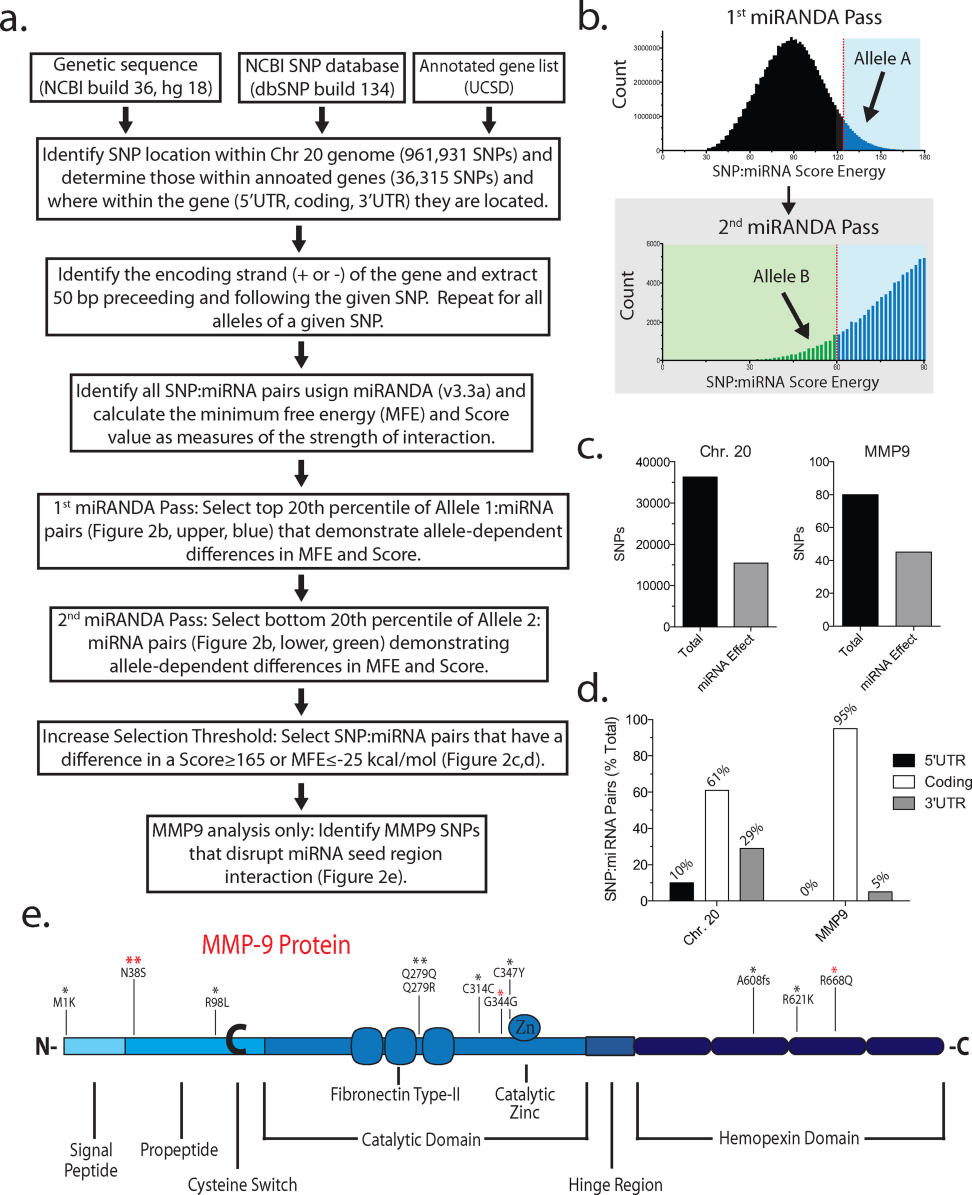
Bioinformatics analysis of allele-dependent alterations in miRNA targeting in chromosome (Chr) 20. (**a**) Bioinformatics work flow for the identification of miRNAs that target SNPs in an allele-dependent manner within Chr 20. (**b**) Distribution of miRNA: allele A Score and Energy (first miRANDA pass). Top 20% of allele A: miRNA pairs (i.e. tightest binding target:miRNA pairs; blue) were chosen for the second miRANDA pass analyzing allele B. Bottom 20% of allele B:miRNA pairs (green) were chosen for further analysis. There were 321 target:miRNA pairs for MMP-9 and 28 494 for Chr 20. The resulting target:miRNA pairs demonstrating SNP-dependent affinity were further selected for pairs that demonstrated large differences in Score (≥165) or Energy (≤−25 kcal/mol) between the two alleles. This ‘high-stringency’ list is shown as Table 1 (MMP-9) or Supplementary Table S2 (Chr 20). The miRNA hybridization to the target was further analyzed by RNAhybrid (http://bibiserv.techfak.uni-bielefeld.de/rnahybrid/) to determine whether the region of hybridization encompasses the polymorphism and whether the polymorphism lies within the seed region. (**c**) Total number of SNPs (black bar) located within Chr 20 or MMP-9 and a subset of SNPs altering the affinity of at least one miRNA (gray bar). (**d**) Locations (5’ or 3’ untranslated region, or coding exon) of SNPs altering miRNA affinity within the gene transcripts of Chr 20 or MMP-9. (**e**) Locations of SNPs within the MMP-9 protein possibly altering miRNA regulation identified by bioinformatics. The miRNA targets most likely biologically active are the subset of all target:miRNA pairs satisfying the selection criteria based on Score and MFE stated above and those where the SNP lies within the presumed seed region of the miRNA (Table 1, underlined). The asterisk (*) indicates the number of miRNAs targeting the given SNP. The four miRNA targets experimentally validated in the present study are indicated by the red asterisk (*).

### Statistical analysis

Individual densitometric, qRT-PCR and ELISA results were presented as mean ± SEM. An unpaired two-sided Student t-test was used for two-group analysis with significance at *P* < 0.05. The luciferase stability assay and qPCR experiments were measured in duplicate and averaged as 1 mean data point; the results were presented as mean ± SEM. For comparison of multiple means, one-way analysis of variance (ANOVA) with a Dunnett or Tukey *post hoc* test was used with a pairwise comparison of the treatment means to the wild-type mean with significance at *P* < 0.05.

## RESULTS

### N-glycosylation deficiency of N38S is not responsible for reduced protein expression

A transient transfection expression of an MMP-9 cDNA harboring the SNP rs41427445 resulted in a profound decrease in the secreted MMP-9 protein in the cell culture supernatant (29). This missense SNP results in the N38S amino acid change; bioinformatics analysis (http://www.cbs.dtu.dk/services/NetNGlyc/) and mass spectrometry analysis have confirmed N38 as one of the two N-glycosylated MMP-9 residues (33,34). A western blot and ELISA analysis of cell culture supernatant of HEK293 cells transfected with N38S–MMP-9 showed a markedly lower amount of secreted protein (Figure 1a), with a corresponding decrease in the enzymatic activity confirmed by gelatin zymography (data not shown). The small amount of expressed N38S–MMP-9 protein was concentrated and, when resolved on an SDS PAGE, demonstrated a mobility shift consistent with the loss of a glycosylation site. This was further confirmed by a selective PNGase removal of the N-glycosylation from the wild-type MMP-9 (Figure 1b), which demonstrated a large mobility shift consistent with the loss of two N-glycosylation sites. In contrast, the N38S showed less mobility shift, consistent with a loss of only one remaining N-glycosylated residue. It is well known that N-glycosylation plays a key role in subcellular protein targeting (35). Our initial hypothesis for the decreased protein secretion of N38S–MMP-9 was that subcellular protein mistargeting and the inability to secrete the protein were due to the loss of N-glycosylation. We tested this hypothesis through immunocytochemistry and a western blot of the cell lysate (i.e. intracellular MMP-9) of the transfected cells expressing the wild type or N38S–MMP-9. We expected increased retention of the mutant protein with detection of mistargeted non-secreted protein in the cells expressing the N38S–MMP-9. In contrast to our expectation, we saw no intracellular accumulation of the mistargeted N38S–MMP-9 protein, but rather a decreased intracellular protein level, confirmed by both immunocytochemistry and the western blot of the whole cell lysate (Figure 1c). We examined for the possibility that the misfolded non-glycosylated N38S protein may have degraded faster, resulting in a lower steady state intracellular protein level, and treated the transfected cells expressing wild type or N38S–MMP-9, with the proteasomal pathway inhibitor MG132 (10 μM) or the autophagic pathway inhibitor chloroquine (50 μg/ml). Inhibition of the proteasomal and autophagic pathway increased the intracellular protein amount of the wild type but did not fully recover the N38S–MMP-9 expression level. This indicated that the loss of N38S protein was not entirely due to accelerated intracellular degradation (chloroquine data not shown) (Figure 1d). Additionally, we attempted to recapitulate an N-glycosylation deficiency of the wild-type MMP-9 with the addition of tunicamycin, which inhibits N-glycosylation, with or without MG132. Tunicamycin treatment of the wild type resulted in a similar reduction of intracellular protein amount comparable to N38S. This Tunicamycin-induced decrease of intracellular wild-type MMP-9 protein was completely rescued by MG132, but the same treatment only partly rescued the N38S protein amount with or without tunicamycin (Figure 1d). The same pharmacological manipulation of the degradation pathways failed to rescue the secreted N38S–MMP-9 protein amounts as well (data not shown). This indicated to us the presence of two cellular mechanisms responsible for the reduced intracellular N38S–MMP-9 protein: (i) a small loss of expression by the degradation of N-glycosylation deficient MMP-9 protein via proteasomal degradation and (ii) an additional, more substantial mechanism at the level of transcription or post-transcription, able to account for the observation that N38S–MMP-9 was not fully rescued by inhibition of the proteasomal or the autophagic pathways.

We next quantified mRNA levels with end-point reverse transcription-polymerase chain reaction (RT-PCR) after transfection of cDNA. We took care to minimize the potential contamination of the analysis from the transfected MMP-9 cDNA by extensive DNase digestion of the extracted total RNA. The level of MMP-9 mRNA was significantly lower for the N38S compared to the wild-type MMP-9-transfected cells despite the use of a cytomegalovirus promoter-driven eukaryotic expression vector in the experiment (Figure 1e). The steady state mRNA level results from a net balance of mRNA transcription and degradation. To assess whether reduction in the N38S–MMP-9 mRNA could be due to altered transcription, wild-type and N38S–MMP-9 cDNA were subcloned upstream of an IRES-EGPF sequence and transfected into HEK293 cells. Since MMP-9 and EGFP are transcribed as a single mRNA in this expression vector, any alteration of transcription by the N38S–MMP-9 insert would be reflected as a reduction in the EGFP expression. Western blots confirmed comparable levels of EGFP expression between the wild-type and N38S–MMP-9-transfected cells; however, again with a reduced expression of N38S–MMP-9 protein compared to wild-type MMP-9 independent of the expression vector used (Supplementary Figure S2), the result suggested a decreased mRNA stability of N38S–MMP-9 mRNA. The stability of MMP-9 mRNA was directly assessed after actinomycin D treatment to inhibit transcription and the mRNA level of N38S–MMP-9 was confirmed to decrease faster with time than the wild-type MMP-9 (Figure 1f). Data suggested a perplexing apparent single nucleotide-dependent difference in mRNA stability leading to a decrease in the mRNA and ultimately the intracellular and extracellular amounts of N38S–MMP-9 protein.

### Bioinformatics analysis of allele-dependent miRNA targets

miRNA degradation of MMP-9 mRNA is a potential mechanism demonstrating such single nucleotide fidelity. We undertook a bioinformatics analysis to identify potential miRNAs targeting the MMP-9 gene. The MMP-9 gene is located on Chr 20 and therefore we restricted our current analysis to only this chromosome. We followed the outline of bioinformatics analysis (Figure 2a) described previously (6) to circumvent the 3’UTR bias of most online web-based programs designed to identify miRNA target sites. The major methodological difference as described by Nicoloso et al. (6) and our present implementation is the number of identified SNPs on Chr 20: it had increased from 78 065 (HapMap Release 21a) in 2010 at the time of the earlier report to 961 931 (NCBI SNP database, version 36.2) at the time of our analysis in 2012. The number of miRNA entries in the database had also increased from 885 to 1919 in the intervening years. Several points are notable: (i) 36 315 SNPs out of a total of 961 931 (∼4%) are located within the 5’UTR, coding exon or 3’UTR of any gene, despite coding exons occupying ∼2% of the genetic space on Chr 20; (ii) 321 (0 in 5’UTR, 312 in coding exon and 9 in 3’UTR) MMP-9 SNPs:miRNA pairs showed allele-dependent difference in minimum free energy (MFE) and Score identified as miRNA:SNP pairs with a threshold of MFE ≤ −16 kcal/mol and Score ≥ 125 for the preferred allele, and MFE ≥ −4 kcal/mol and Score ≤ 60 for the less tightly bound allele (Figure 2b); (iii) the wild-type or polymorphic allele can be preferentially targeted by miRNA, resulting in loss-of-regulation or gain-of-regulation of the SNP, respectively; (iv) a given SNP can be targeted by several different miRNAs; and (v) several SNPs resulting in no amino acid changes (i.e. synonymous mutations) are discernible by the targeting miRNAs. A more stringent list of MMP-9 SNP:miRNA pairs selected by ΔMFE ≤ −25 kcal/mol or ΔScore ≥ 165 difference between the alleles is shown on Table 1. miRNAs are thought to mostly target the 3’UTR to regulate gene expression; therefore, most available bioinformatics tools designed to identify potential miRNA targets for any given gene focus on the 3’UTR. Our bioinformatics approach did not have this 3’UTR bias, but searched the entire Chr 20 for SNP-dependent differential miRNA hybridization and identified several miRNAs targeting the MMP-9 coding exon, including ones targeting the N38S SNP. MMP-9 SNPs disrupting miRNA seed regions were determined (Table 1, underlined) and chosen for downstream experimental validation (Figure 2e). For MMP-9, 95% of SNP-dependent miRNA targets were found in the coding exon with a small number of targets in the 3’UTR. Likewise, generalization to Chr 20 suggested the predominance of SNPs in the coding exon as targets followed by 3’UTR and 5’UTR (Figure 2c and d). Next, we sought experimental confirmation of selected SNP-dependent miRNA-mediated regulation of MMP-9 at the mRNA and protein levels.

**Table 1.** SNP-dependent miRNA targets in the MMP-9 transcript

ID	rs#	miRNA	Targeted allele	ΔScore	ΔMFE
Met1Lys	rs121434556	hsa-miR-4498	T (WT)	179	−26.73
Met1Lys	rs121434556	hsa-miR-637	T (WT)	165	−28.35
Met1Lys	rs121434556	hsa-miR-4446-3p	T (WT)	163	−25.07
Met1Lys	rs121434556	hsa-miR-1909-3p	T (WT)	154	−26.95
Met1Lys	rs121434556	hsa-miR-4656	A (SNP)	147	−26.64
Asn38Ser	rs41427445	**hsa-miR-671-3p**	G (SNP)	199	−26.6
Asn38Ser	rs41427445	hsa-miR-1301-3p	G (SNP)	189	−26.71
Asn38Ser	rs41427445	hsa-miR-22-3p	G (SNP)	184	−17.96
Asn38Ser	rs41427445	**hsa-miR-657**	G (SNP)	172	−18.12
Thr40=	rs139837207	hsa-miR-4727-5p	T (SNP)	210	−24.92
Gln43His	rs142807270	hsa-miR-3127-3p	G (WT)	172	−25.72
Gln43His	rs142807270	hsa-miR-1260b	G (WT)	169	−22.79
Arg98Leu	rs140352541	hsa-miR-762	G (WT)	167	−27.46
Asn127Lys	rs3918252	hsa-miR-3921	G (SNP)	168	−15.16
Asn127Lys	rs3918252	hsa-miR-4684-3p	G (SNP)	164	−16.55
Arg239His	rs28763886	hsa-miR-449c-5p	G (WT)	179	−23.05
Glu274Asp	rs141318648	hsa-miR-1233	G (WT)	165	−19.31
Glu274Asp	rs141318648	hsa-miR-611	C (SNP)	126	−26.45
Gln279Arg	rs17576	hsa-miR-3934-5p	G (SNP)	167	−20.99
Gln279=	rs139262778	hsa-miR-4685-5p	G (WT)	131	−27.99
Gln279Arg	rs17576	hsa-miR-1247-5p	G (SNP)	128	−25.24
Cys302Ter	rs146499495	hsa-miR-369-5p	A (SNP)	169	−15.74
Cys302Ter	rs146499495	hsa-miR-4479	C (WT)	168	−22.03
Cys314=	rs139509848	hsa-miR-4726-5p	C (WT)	143	−25.91
Gly344=	rs138668828	**hsa-miR-4707-5p**	G (WT)	125	−25.32
Cys347Tyr	rs145450508	hsa-miR-3714	G (WT)	133	−25.2
Pro459Ser	rs147087483	hsa-miR-4723-5p	T (SNP)	185	−23.76
Ala525=	rs80077409	hsa-miR-671-3p	G (WT)	165	−20.22
Asp558Gly	rs150224695	hsa-miR-608	G (SNP)	165	−24.19
Pro561Ser	rs141966515	hsa-miR-663a	T (SNP)	166	−34.67
Phe571Val	rs35691798	hsa-miR-5192	G (SNP)	172	−24.82
Ala608fs	rs34003985	hsa-miR-4787-3p	- (SNP)	144	−25.56
Asp608fs	rs34003985	hsa-miR-668	- (SNP)	167	−31.53
Arg621Lys	rs6104428	hsa-miR-3194-5p	G (WT)	143	−27.06
Arg668Gln	rs17577	**hsa-miR-4783-3p**	G (WT)	171	−25.2
Arg668Gln	rs17577	hsa-miR-4763-5p	C (SNP)	142	−26.49
V682M	rs137904662	hsa-miR-28-5p	G (WT)	174	−15.99
Ser686Asn	rs151173908	hsa-miR-133a	G (WT)	167	−21.37
Ser686Asn	rs151173908	hsa-miR-133b	G (WT)	167	−21.37
3'UTR-C3T	rs20544	hsa-miR-4673	T (SNP)	165	−19.64
3'UTR-A39C	rs1056628	hsa-miR-4746-5p	C (SNP)	185	−20.13

**Figure 3.**
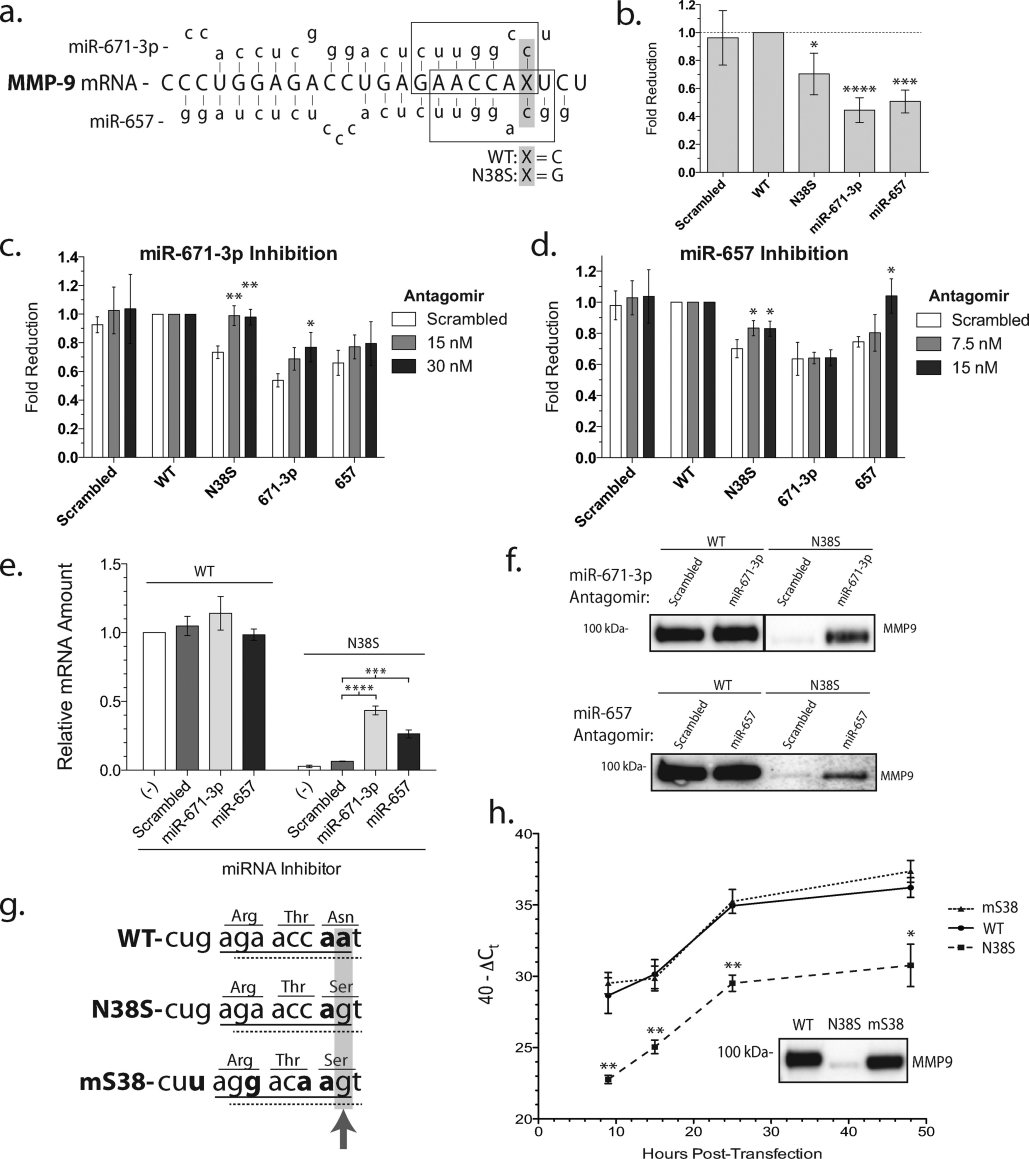
Selective targeting of the N38S allele by miRNAs decreases MMP-9 mRNA and protein expression. (**a**) Cartoon illustration of miR-671**-**3p and miR-657 targeting MMP-9 mRNA. Boxes represent miRNA seed regions; the gray-highlighted region with ‘x’ represents the location and nucleotide of the N38S polymorphism. (**b**) Luciferase stability after the insertion of a tandem MMP-9 coding sequence of either the wild type (WT) or N38S–MMP-9 sequence in the 3’UTR of firefly luciferase. The N38S–MMP-9 sequence resulted in a reduction in stability compared to the wild-type-MMP-9 sequence. *P< 0.05, ***P < 0.0005, ****P < 0.0001, ANOVA and *n* = 4. (**c**) Luciferase stability after the addition of miR-671-3p antagomirs. *P < 0.05, **P < 0.001, ANOVA and *n* = 3. (**d**) Luciferase stability after the addition of miR-657 antagomirs. *P < 0.05, ANOVA and *n* = 3. (**e**) Relative mRNA levels (mean ± SEM) of WT or N38S–MMP-9 after no treatment or treatment with scrambled or miRNA antagomirs [miR-671-3p (30 nM) and miR-657 (15 nM) for 36 h]. ***P < 0.0005, ****P < 0.0001, ANOVA and *n* = 3. (**f**) A representative western blot of secreted MMP-9 in cell culture media from the above miRNA inhibitor experiment. (**g**) Silent miRNA binding site mutagenesis strategy. Underlined regions represent the seed region binding sites of miR-671**-**3p (solid) or miR-657 (dashed). The gray-highlighted region and arrow indicate the site of the N38S polymorphism and the bold text indicates target:miRNA base-pair mismatch. Note the additional three base mismatches engineered into the mS38 while preserving the encoded amino acids. (**h**) qRT-PCR time course of MMP-9 mRNA after transfection of the respective plasmids. 40−Δ*C*_t_ represents the total mRNA amount and plotted as mean ± SEM from three independent experiments. *P < 0.05, **P < 0.001 and ANOVA. Inset: western blot data of the MMP-9 protein in cell media at 24-h post-transfection.

MMP-9 polymorphisms displaying allele-dependent alterations in miRNA affinity are tabulated. ID indicates the residue and the resulting change in the amino acid encoded by the polymorphism where =: synonymous; fs: frame shift; 3’UTR: 3’ untranslated region. The rs# is the SNP reference number and the miRNAs (human) targeting that SNP are listed. The targeted allele represents the polymorphic nucleotide that is preferentially targeted; miRNA can target the wild-type or the SNP allele. If an miRNA targets the wild-type allele, the SNP allele escapes miRNA regulation with a likely increase in expression and vice versa for the miRNA targeting the SNP allele. ΔScore and ΔMFE indicate the difference in the Score and MFE between the two alleles. The target:miRNA ‘high-stringency’ pairs were selected for ΔScore ≥ 165 or ΔMFE ≤ −25 kcal/mol from a less stringent list of target:miRNA pairs identified by the bioinformatics algorithm outlined in Figure 2. Underlined text represents SNPs located within the predicted miRNA binding seed region (locations of SNPs within MMP-9 are represented in Figure 2e) and the bold text indicates experimentally validated results in the current study. Arg668Gln targeted by miR-326, showing no statistical effect (Figure 3a and b), was not included in this table because ΔScore (141) and ΔMFE (−22.1 kcal/mol) did not meet the high-stringency selection criteria.

### Dual miRNA allele-specific targeting of N38S–MMP-9 transcript

We first focused on miRNAs targeting the N38S polymorphism (see Table 1). The four miRNAs identified as potentially demonstrating N38S polymorphism-specific binding (miR-671-3p, miR-1301-3p, miR-22-3p and miR-657) all had higher affinity toward the N38S polymorphism compared to the wild-type allele. Of these four miRNAs, only the miR-671-3p and miR-657 seed region target sequences were affected by the N38S polymorphism. As expected for miRNAs where the polymorphism is outside the seed region, miRNA inhibitor studies using miR-1301-3p or miR-22-3p antagomirs failed to rescue N38S–MMP-9-secreted protein levels (Supplementary Figure S3). Alternatively, the failure of these antagomirs to rescue the N38S–MMP-9 expression could indicate very low levels of miR-1301-3p and miR-22-3p rendering these miRNAs biologically irrelevant in our model system.

We next focused on miR-671-3p and miR-657 that target the nucleotides encompassing the N38S codon. The miR-671-3p is one of the two mature miRNAs derived from a larger 118-bp pre-miR-671 (NR_030407). miR-671 has been reported to target the cerebellar degeneration-related protein 1 (36) and is differentially regulated in bone marrow samples from patients with myelodysplastic syndromes (37). miR-657 targets the 3’UTR of transducing like enhancer protein 1 and has been reported to play a role in hepatocellular tumorigenesis (38). It also targets the 3’UTR of insulin-like growth factor in an insertion/deletion polymorphism specific manner (39). However, to our knowledge, there is no prior report of either miRNAs targeting MMP-9. Sequence alignment indicated that the mature miR-671-3p and miR-657 both matched the core seed region, 7–8 nucleotide sequence encompassing the N38S polymorphism, but it did not match at the critical +1 or +2 nucleotide location for the wild type, respectively (Figure 3a). Both miR-671-3p and miR-657 were detected in HEK293 cells using qRT-PCR capable of amplifying small RNA (Supplementary Figure S4). To investigate the N38S–MMP-9 polymorphic instability, a tandem repeat of wild type or N38S–MMP-9 coding sequence was inserted into the 3’UTR of a firefly luciferase gene (fluc-wt-MMP-9 or fluc-N38S–MMP-9), creating miRNA binding site reporters as described previously (40,41). A tandem scrambled sequence lacking known miRNA recognition and tandem miR-671-3p or miR-657 complementary targeting sequences were also inserted into the 3’UTR of firefly luciferase creating a negative- and two positive-control fluc-miRNA reporters. Both positive-control reporters fluc-miR-671-3p and fluc-miR-657 displayed the greatest level of fold reduction in the luciferase signal compared to fluc-wt-MMP-9, while fluc-N38S–MMP-9 showed an intermediate but significant reduction in luciferase stability. The negative control fluc-scrambled reporter showed no significant change in the luciferase signal (Figure 3b). qRT-PCR quantitation of luciferase mRNA gave results identical to the enzymatic assay (Supplementary Figure S5). The data validate the fluc-miRNA reporter constructs and confirm differential mRNA instability conferred by the polymorphic MMP-9 coding sequences about the N38S site.

If the polymorphic MMP-9 coding sequence about the N38S site, as reported by the fluc-miRNA reporter is due to endogenous miRNA binding to the polymorphic sequence, the inhibition of such endogenous miRNA should recover the luciferase signal. Antagomirs (miRNA silencing anti-sense oligonucleotides) were used to investigate the roles of miR-671-3p and miR-657 in fluc-N38S–MMP-9 instability. First, we confirmed that the small molecule miRNA antagomirs do indeed specifically reduce the endogenous levels of target miRNA (Supplementary Figure S4). Next, we documented that the addition of the miR-671-3p antagomir resulted in a complete recovery, while adding miR-657 antagomir resulted in a partial but significant increase in the fluc-N38S–MMP-9 signal with respect to the scrambled antagomir control (Figure 3c and d). The respective antagomirs demonstrated the expected and specific increase in the fluc-miRNA reporter signals, although the miR-671-3p antagomir trended toward a concentration-dependent recovery of the fluc-miR-657 signal (i.e. cross-reactivity) which was not statistically significant. These results suggested that miR-671-3p plays a more prominent role than miR-657 in regulating the N38S–MMP-9 mRNA stability.

To determine the effects of miR-671-3p and miR-657 on MMP-9 rather than the fluc-miRNA reporters, cells expressing the full-length wild-type or N38S cDNAs were co-transfected with the miRNA antagomirs and the MMP-9 mRNA amounts quantified by qRT-PCR. The miRNA antagomirs for either miR-671-3p or miR-657 did not change the mRNA abundance compared to the scrambled control for the wild-type MMP-9 but significantly increased the mRNA level of N38S (Figure 3e). A western blot of the miRNA antagomir-treated cells confirmed the expected increase in secreted N38S-SNP protein, while having no effect on the wild-type protein expression (Figure 3f). Similar results were observed for the intracellular rescue of N38S–MMP-9 protein levels compared to wild-type MMP-9 protein (data not shown). These observations are consistent with the idea that endogenous miR-671-3p and miR-657 target the MMP-9 mRNA in an SNP-specific manner. Interestingly, the rescue of the N38S mRNA and protein was incomplete suggesting an incomplete inhibition of the targeted miR-671-3p by the antagomirs (Figure 3c), possibly due to the limitation of 30-nM antagomir that can be used without non-specific inhibition of the protein expression (see the Materials and Methods section). Likewise, we were unable to perform the dual inhibition of miR-671-3p and miR-657 experiment because of the concentration-dependent non-specific effect of these small molecule reagents.

To confirm the role of miRNA targeting the codons surrounding the N38S SNP in MMP-9 mRNA regulation, an additional approach was taken by introducing artificial codon changes that disrupted potential miRNA binding stability while preserving the amino acid sequence. The mutant-S38 sequence preserved the N38S amino acid change while disrupting both the miR-671-3p and miR-657 recognition sequence in the critical nucleotides 2–8 seed region (Figure 3g). We named this construct harboring the silent nucleotide changes mS38. qRT-PCR quantitation of MMP-9 mRNA levels at different time points after transfection demonstrated consistently lower levels of mRNA for the N38S-SNP compared to the wild type, and a complete recovery of wild-type level mRNA abundance for the mS38-harboring cells was observed (Figure 3h). A western blot analysis of wild-type, N38S and mS38-transfected cells showed a complete recovery of extracellular mS38 protein despite the lack of N-glycosylation at residue 38. (Figure 3h, inset).

### miRNA differential targeting of R668Q-MMP-9

**Figure 4.**
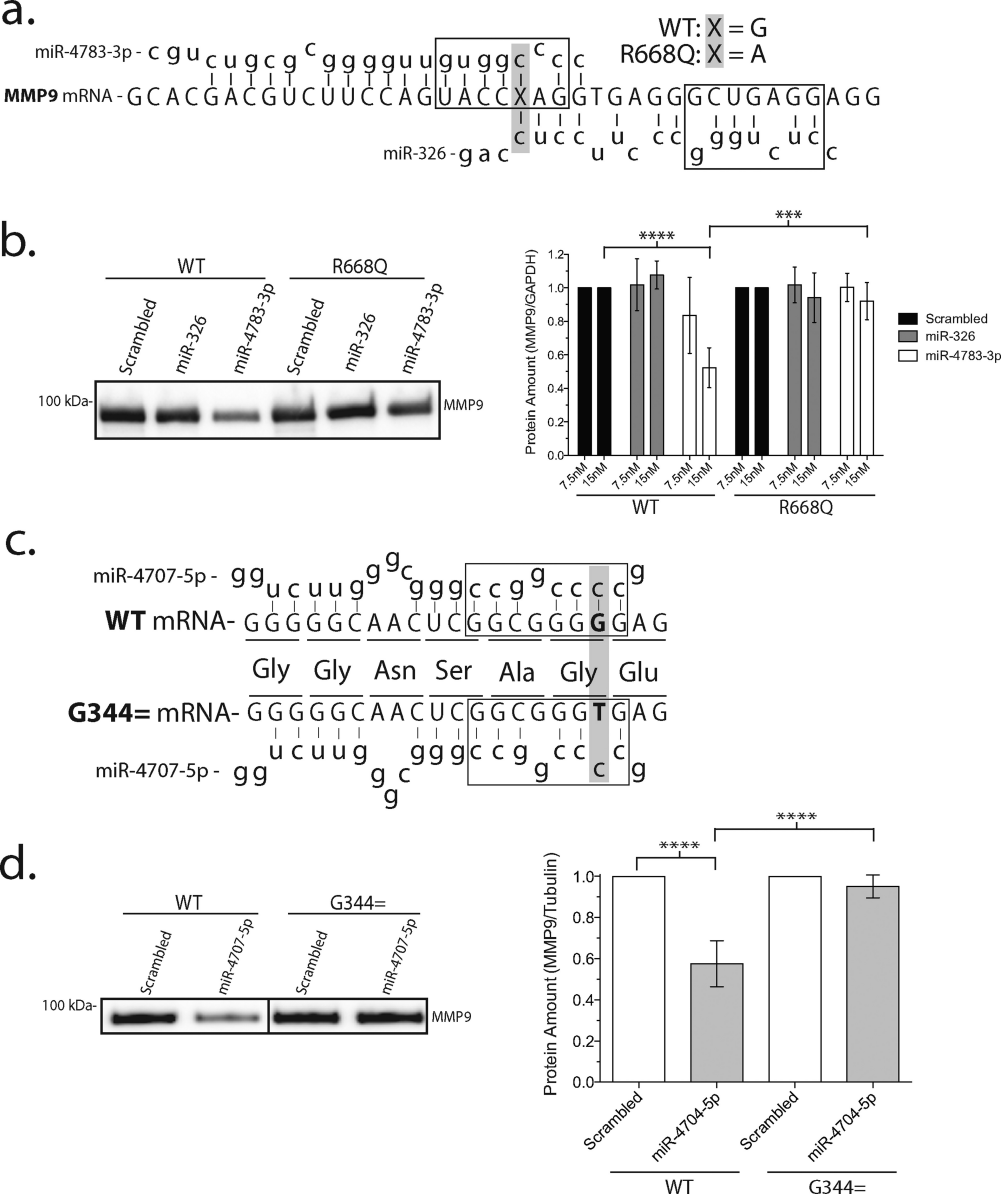
Additional miRNAs preferentially targeting the wild-type MMP-9 coding exon. (**a**) Cartoon illustration of miR-4783**-**3p and miR-326 targeting MMP-9 mRNA. Boxes represent the miRNA seed region and the gray-highlighted region with ‘x’ represents location and nucleotide of the R668Q polymorphism. Note that the polymorphism is within the seed region for miR-4783-3p but outside for miR-326. Additionally, miR-326 failed to meet the inclusion criteria based on ΔScore and ΔMFE from the bioinformatics inquiry. (**b**) Western blot of extracellular wild type or R668Q-MMP-9 protein in cells treated with 15-nM miRNA mimics. Densitometric quantification of wild type or R668Q-MMP-9 protein (mean ± SEM) after addition of 7.5-nM or 15-nM miR-326 or miR-4783-3p mimics. ***P < 0.0005, ****P < 0.0001, ANOVA and *n* = 4. (**c**) Cartoon illustration of miR-4707-5p targeting wild type or G344 = (synonymous SNP) MMP-9 mRNA. Boxes represent the miRNA seed region and the gray-highlighted bold text indicates the site of the synonymous SNP. Codon (underlined text) and corresponding amino acid sequence is given. (**d**) Western blot of wild type or G344 = MMP-9 protein (mean ± SEM) after treatment with 10-nM miRNA mimics for 36 h. Densitometric quantification of western blots. ****P < 0.0001, ANOVA and *n* = 4.

The precise mechanism of miRNA targeting to mRNA is unknown, but the critical role of the 8 nucleotide seed region on the 5’ end of the miR:target complex has emerged (2). We tested the importance of the seed region recognition in miRNA inhibition of polymorphic MMP-9 mRNA by examining two miRNAs predicted by bioinformatics to target the R668Q-SNP in the MMP-9 coding exon (rs17577), but with miR-326 predicted to hybridize outside and miR-4783-3p within the seed region (Figure 4a). Both miRNAs are predicted to preferentially inhibit the wild-type G allele over the SNP A/C allele. Western blot for secreted protein amount (Figure 4b) demonstrated the expected concentration-dependent reduction in the wild-type (G allele) protein by administration of miR-4783-3p miRNA mimic (oligonucleotide miRNA mimic) while having little effect on the R668Q (A allele). miR-326 that targets the same area of the MMP-9 exon, but where the R668Q-SNP lies outside the critical seed region, did not affect the protein amounts. Similar observations were confirmed for the intracellular protein amounts obtained from the cell lysate (data not shown). Control experiments with these miRNA mimics confirmed the expected increase in the respective miRNAs detected by qRT-PCR (Supplementary Figure S6). These data, in addition to the earlier observation that miR-1301-3p and miR-22-3p targeting the non-seed region of MMP-9 near the N38S polymorphism did not affect expression (Supplementary Figure S3), suggested the possible importance of disrupting the miRNA seed region binding for the manifestation of polymorphism-specific miRNA regulation. However, unlike the stringent perfect match required for the siRNA binding to the mRNA target, miRNA binding its target mRNA tolerates mismatch with 60% of the seed interactions being noncanonical with bulges and mismatches (42). Therefore sequences outside of the seed region are likely to affect the miRNA interactions with SNP-harboring mRNA targets.

### Discrimination of synonymous SNPs by miRNA

Lastly, synonymous SNPs, in which no change in the encoded amino acid is introduced by the nucleotide change, could be susceptible to SNP-dependent miRNA targeting. cDNAs encoding the synonymous SNPs (C314, rs139509848; Gln279, rs139262778; Gly344, rs138668828) targeted by miR-4726-5p, miR-4685-5p and miR-4707-5p, respectively, where the nucleotide altered by the synonymous SNP falls within the miRNA target seed region (Table 1), are likely SNP-dependent targets. We created a cDNA encoding the Gly344 (Figure 4c) and co-transfected HEK293 cells with the miR-4707-5p miRNA mimic. This miRNA is predicted to show greater affinity to the wild type compared to the Gly344 mRNA and we predicted the miR-4707-5p miRNA-mimic treatment to show inhibition of the wild type but not Gly344 mutant protein. Quantitation of the extracellular protein secreted confirmed a reduced level of Gly344 mutant compared to the wild type (Figure 4d), consistent with the idea that synonymous SNPs are also targets of SNP-dependent regulation by miRNA.

## DISCUSSION

Transcriptional regulation of MMP-9 by cytokines and developmental queues is considered to be the predominant mechanism of controlling expression of this pleiotropic molecule (27). Two recent reports indicate that miRNA may play a role in regulating the expression of MMP-9. Nothnick et al. (43) demonstrated that of the many miRNAs differentially expressed in the uterus in response to estrogen treatment, bioinformatics analysis indicated that miR-705 binds to the 3’UTR of the MMP-9 mRNA. In another recent case: control analysis of miRNA differentially expressed in human subjects with thoracic aortic aneurysm identified miR-29 targeting a 3’UTR of the MMP-2 gene. The authors demonstrated that an *in vitro* over-expression of miR-29 downregulated the expression of MMP-2 mRNA, directly confirming the role of miRNA in regulating MMP expression (44). However, an SNP-specific regulation of MMP-9 by miRNA targeting the coding exon has not been reported.

Our results showed an active miRNA-mediated post-transcriptional regulation of MMP-9 targeting the coding exon in an SNP-specific manner adding an additional layer of complexity to the regulation of MMP-9. We observed a profound decrease in extracellular expression of the MMP-9 protein when cDNA engineered with the naturally occurring N38S SNP was transfected into HEK293 cells. This specific missense SNP altered the N-glycosylation post-translational modification of MMP-9 with potential effects on sub-cellular protein targeting, folding and degradation. However, a systematic study of the reason for the reduced mutant SNP-harboring protein expression led us to the conclusion that the regulation was at the level of MMP-9 mRNA and not post-translational due to the loss of N-glycosylation. The strongest evidence for this surprising conclusion was the complete rescue of the mRNA as well as secreted protein amounts in an engineered mutant mS38 that retained the amino acid change from asparagine to serine at this residue, retaining an N-glycosylation deficiency, but altering the miR-671-3p and miR-657 recognition sequences. Further experiments with fluc-miRNA reporters and miRNA antagomirs gave results consistent with a selective targeting of the N38S SNP mRNA while sparing the wild type. The incomplete rescue of N38S–MMP-9 mRNA and protein levels comparable to wild-type MMP-9 suggested two possibilities: (i) failure to completely inhibit endogenous miR-671-3p and miR-657 simultaneously or (ii) the presence of novel miRNA acting in an N38S SNP-dependent manner not currently recognized and included in our bioinformatics analysis.

Our results show that the same miRNA binding sequence in the MMP-9 coding exon when placed in the 3’UTR of the luciferase (i.e. fluc-miRNA reporter) had a diminished silencing effect (compare Figure 1a and e, ∼70% inhibition to Figure 3b and c, only 30% inhibition). Our fluc-miRNA reporter had two copies of the miRNA target sequence introduced into the 3’UTR of the luciferase vector but was devoid of the surrounding endogenous sequences. It is thought that endogenous miRNA recognizes not only the exact match in the seed region but also mRNA sequence upstream; thus, the surrounding nucleotide sequences also affect the binding of endogenous miRNA leading to the concept of noncanonical target recognition by miRNA [(17); also see reviews by Jackson & Standart (42) and Pillai et al. (45)]. Furthermore, whether the miRNA and target interaction has base mismatches influences the mechanism of inhibition by altering the mRNA stability versus inhibiting translational efficiency. Another interesting mechanism whereby sequences near the miRNA binding site can influence miRNA effect was reported by Elcheva et al. (46). They reported that a sequence-specific protein binding near an miRNA target affects miRNA binding to its site through steric hindrance. Such a mechanism could also explain the quantitative discrepancy between miRNA effects on endogenous site and sequence introduced into a luciferase reporter. Therefore, while the fluc-miRNA reporter is a convenient and widely utilized tool for identifying putative miRNA binding sequences, we believe it is not an exact replication of the miRNA interaction with mRNA target and consequently differs in the quantitative amounts of inhibition observed. Nevertheless, our experimental evidence suggests the importance of the seed region between the miRNA and MMP-9 mRNA target interaction. Experimental evidence also confirmed the presence of multiple miRNAs targeting the same mRNA site in an SNP-specific manner and miRNA's ability to recognize synonymous SNPs with no change in the encoded amino acid, hitherto considered a silent mutation with no biological consequences.

We wondered whether the SNP-specific regulation by miRNA was prevalent. At the genome-wide level, a preliminary search of the miRanda database (http://www.mirbase.org/index.shtml) listed 1100 human miRNAs with over 16 million putative target DNA sites in the human genome. This is considered an underestimation due to the 3’UTR bias of most miRNA target search algorithms. We undertook a bioinformatics task to map an unbiased miRNA target hit list with genome-wide SNP data, asking whether other miRNAs differentially target an SNP locus regardless of whether the site is in the coding exon or in the 5’ or 3’UTR. For MMP-9, the initial selection was based on a large difference in the SNP-dependent change in the Score and MFE (≥125 Score and ≤−16 kcal/mol for the preferred binding genotype and ≤60 Score and ≥−4 kcal/mol for the weaker binding genotype), corresponding to a change in the miR:target interaction strength from the top 20% for the preferred allele to the bottom 20% for the other allele yielded 321 miRNA:target pairs with an overwhelming occurrence in the coding exons (Figure 2d). After selection for high-stringency miRNA:target pairs (ΔScore ≥ 165 or ΔMFE ≤ −25 kcal/mol) and determination of whether the polymorphism resulted in a disruption of the miRNA seed region (Table 1, underlined), we experimentally validated four of the miRNAs predicted to show an SNP-dependent effect on MMP-9 expression. We believe the high-stringency selection of miRNA:target pairs identified by our bioinformatics analysis tended to select for miRNA hybridizing to the SNP-containing seed region with the highest likelihood of being biologically active; however, the biological validity of the SNP-dependent miRNA:target pairs identified by bioinformatics must be experimentally confirmed. Chr 20, where the MMP-9 gene resides, consists of ∼63 million bp encoding 654 genes (University of California Santa Cruz annotation July 2012). Extension of the bioinformatics analysis to Chr 20 indicated a large number (15 359; Supplementary Table S2) of SNP-specific miRNA:target pairs that exhibited a large allele-specific difference in binding, with ∼61% targeting a coding exon (Figure 2d) covering 521 genes. It is thought that miRNAs regulate gene expression by mostly targeting the non-coding 3’UTR of mRNA, but we add to the increasing evidence indicating that the coding sequence may be targeted, perhaps even predominantly, by miRNA (18,47). Our analysis of Chr 20 suggested the generality of the SNP-specific regulation of gene expression by miRNA targeting the coding exon experimentally confirmed for MMP-9. The unbiased strategy for identifying SNP-dependent miRNA:target pairs could serve as a road map for a systematic discovery of SNP–miRNA interactions likely to be abundant in the human genome. Prior bioinformatics and experimental reports have suggested the presence of polymorphism in the miRNA targets in the 3’UTR (12–16) and increasing evidence supports the coding exon as a likely target for miRNA (17–19). Our work is the first to combine these observations to demonstrate that SNPs within the coding exons strongly influence the miRNA regulation of target gene expression.

Tight regulation of the multi-functional MMP-9 is critical for the normal function of the organism. miRNA suppression of a given coding region SNP through specific targeting of the affected nucleotide is a novel cellular mechanism of gene regulation with broad implications in pathogenesis of diseases, biomarker studies seeking association between MMP-9 and diseases and in basic cell biology. We believe the characterization of the novel regulatory mechanism of MMP-9 expression demonstrated here will impact a wide range of normal biology and diseases in which MMP-9 plays a fundamental role. It is estimated that 37 million genetic variants exist in the human genome, including structural variants such as deletions and copy number variants, with the majority being SNPs. Focused exome sequencing leveraging the advances in sequencing technology has allowed identification of rare SNPs contributing to Mendelian diseases and is likely to lead to further discoveries on the role of genetic variants in multifactorial complex diseases (48,49). An SNP-specific miRNA regulation of gene expression is likely to be prevalent; tissue specific miRNA expression and the consequences of such regulation must be considered to better understand the impact of a given SNP of the exome on normal biology and pathoetiology.

## ACCESSION NUMBER

BC006093

## SUPPLEMENTARY DATA

Supplementary Data are available at NAR Online.

SUPPLEMENTARY DATA
