# Supplementary material for: Single nucleotide polymorphism-specific regulation of matrix metalloproteinase-9 by multiple miRNAs targeting the coding exon
Source: Nucleic Acids Res. 2014 Mar 13;42(9):5518–31. doi: 10.1093/nar/gku197 (PMC4027190; doi:10.1093/nar/gku197)
Supplement: SUPPLEMENTARY DATA [file supp_42_9_5518__index.html]

Single nucleotide polymorphism-specific regulation of matrix metalloproteinase-9 by multiple miRNAs targeting the coding exon — SUPPLEMENTARY DATA 

# Single nucleotide polymorphism-specific regulation of matrix metalloproteinase-9 by multiple miRNAs targeting the coding exon

## SUPPLEMENTARY DATA

**Files in this Data Supplement:**

- Supplemental Figures
- Supplemental Figures
