# Supplementary material for: Single nucleotide polymorphism-specific regulation of matrix metalloproteinase-9 by multiple miRNAs targeting the coding exon
Source: Nucleic Acids Res. 2014 Mar 13;42(9):5518–31. doi: 10.1093/nar/gku197 (PMC4027190; doi:10.1093/nar/gku197)
Supplement: SUPPLEMENTARY DATA [file supp_gku197_nar-00006-y-2014-File007.pdf]

Single nucleotide polymorphism-specific regulation of matrix metalloproteinase-9 by multiple miRNAs targeting the coding exon

Supplementary Materials

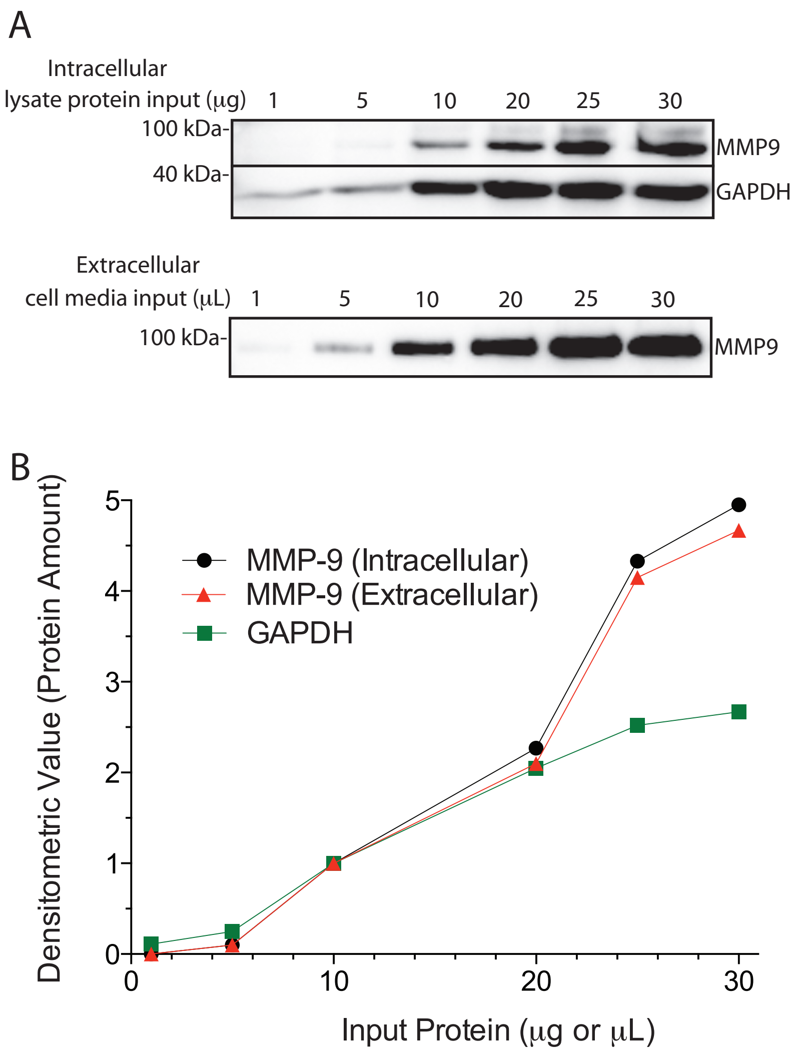

Supplemental Figure 1: Duellman et al.

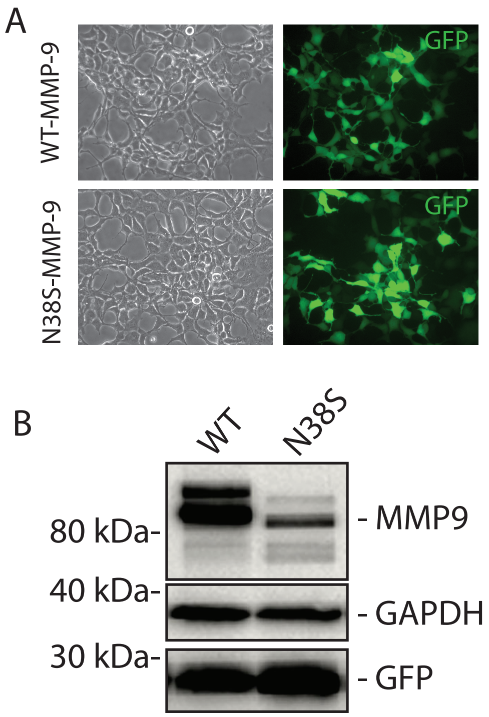

Supplemental Figure 2: Duellman et al.

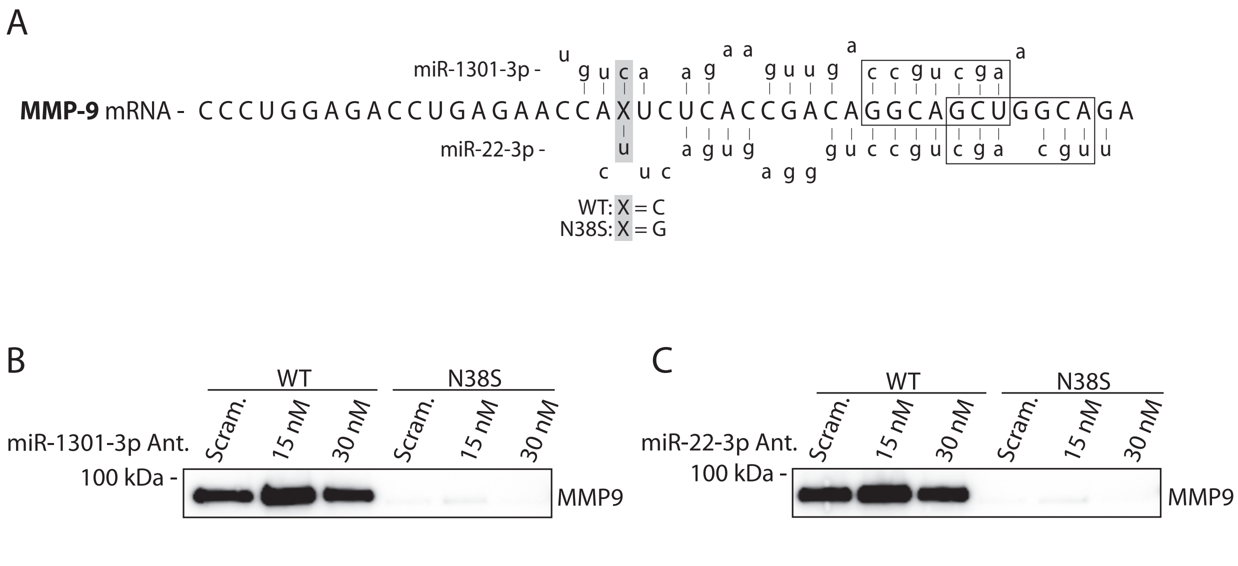

Supplemental Figure 3: Duellman et al.

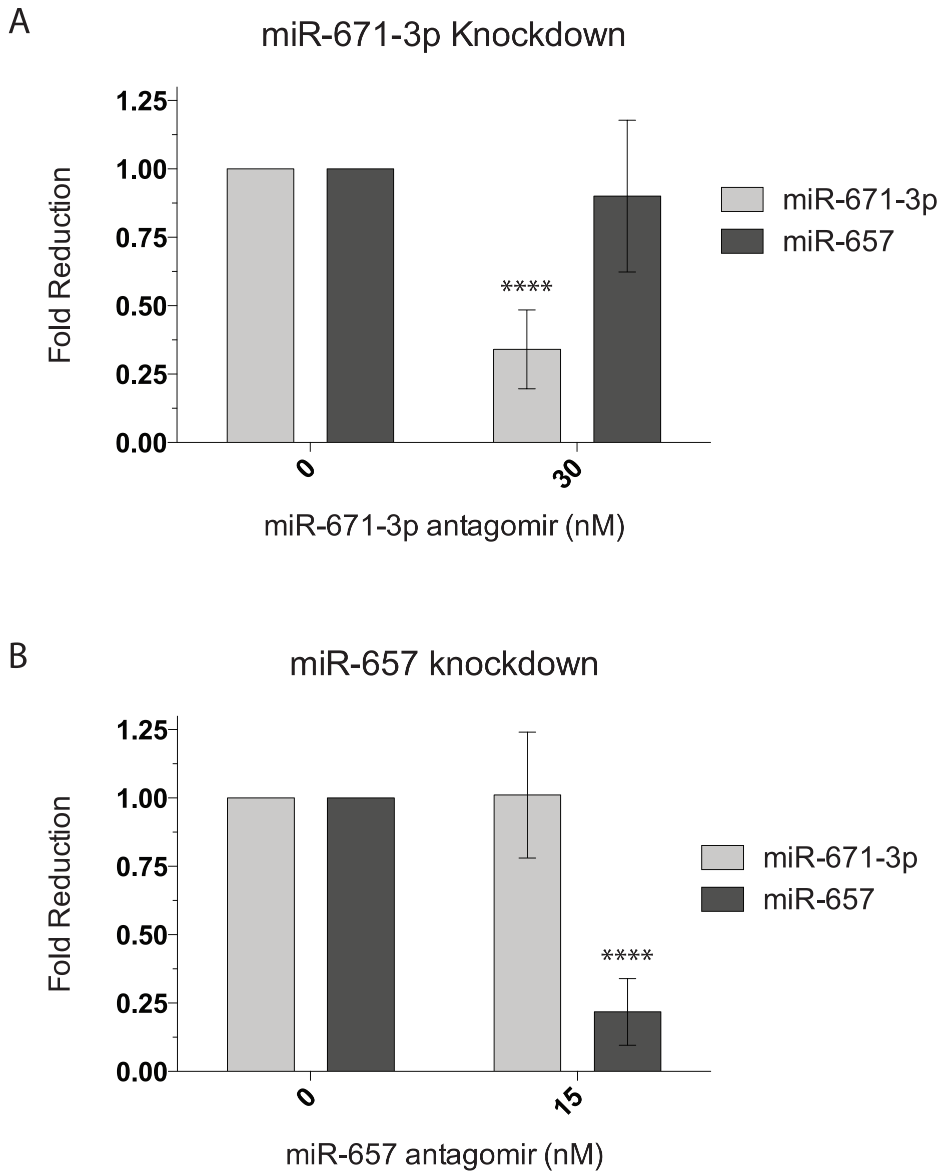

Supplemental Figure 4: Duellman et al.

A

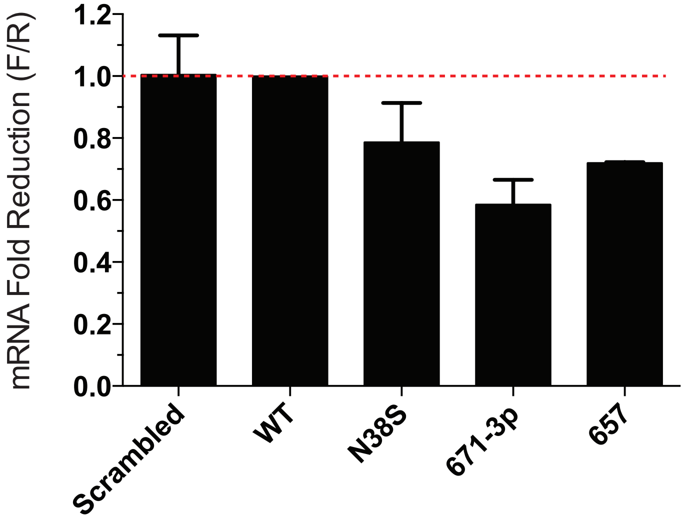

Supplemental Figure 5: Duellman et al.

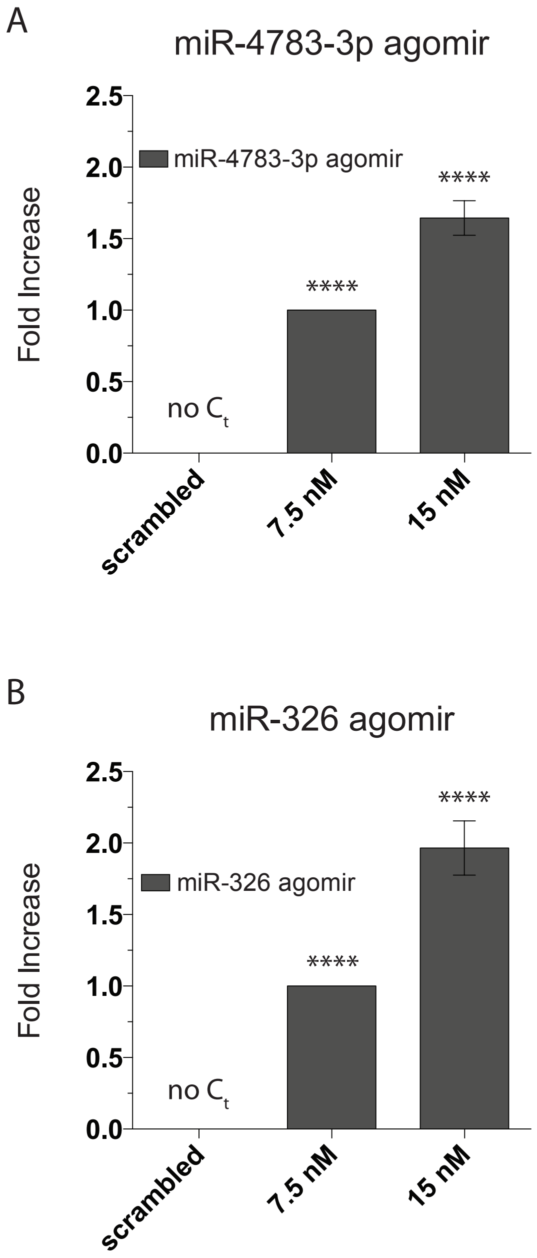

Supplemental Figure 6: Duellman et al.

Supplemental Table 1: Oligonucleotides used for MMP9 studies

Mutagenesis:

MMP9 *External Primers*

Fwd Primer: TCACCATGAGCCTCTGGCAG

Rev Primer: GAGCCCTAGTCCTCAGGGCAC

N38S *rs41427445 (A>G SNP)*

Fwd Primer: ATGAGCCTCTGGCAGCCCCTGGTCCTGGTGCTCCTGGTGCTGGGCTGCTGC TTTGCTGCCCCCAGACAGCGCCAGTCCACCCTTGTGCTCTTCCCTGGAGACCTGAGAACCAGTCTC ACCGACAGGCAG

mS38 *silent miRNA binding site mutagenesis*

Fwd Primer: ATGAGCCTCTGGCAGCCCCTGGTCCTGGTGCTCCTGGTGCTGGGCTGCTGC TTTGCTGCCCCCAGACAGCGCCAGTCCACCCTTGTGCTCTTCCCTGGCGATCTTAGGACAAGTCTC ACCGACAGGCAG

R668Q *rs17577 (G>A SNP)*

Fwd Primer: GTCTTCCAGTACCAAGAGAAAGCCTATTTCTGCCAGGACCGC

Rev Primer: ATAGGCTTTCTCTTGGTACTGGAAGACGTCGTGCGTGTCCAA

G344= *rs138668828 (G>T SNP)*

Fwd Primer: TGGGGGGCAACTCGGCGGGTGAGCTGTGCGTCTTCCCCTTCACTTTCC

Rev Primer: GCACAGCTCACCCGCCGAGTTGCCCCCCATCACC

qRT-PCR:

MMP9

Fwd Primer: TCACCATGAGCCTCTGGCAG

Rev Primer: CTCACCGGTCTTGGGCAGGGACAGTTGCTTC

Firefly Luciferase

Fwd Primer: TCTGAGGAGCCTTCAGGATT

Rev Primer: AGATGGAACCTCTTGGCAAC

Renilla Luciferase

Fwd Primer: AAGAGCGAAGAGGGCGAGAA

Rev Primer: TGCGGACAATCTGGACGAC

GAPDH

Qiagen Lot #PPH00150F

miRNA detection – Taqman MicroRNA Assay, Life Technologies

miR-326

PN4427975: RT000542, TM000542

miR-657

PN4427975: RT001512, TM001512

miR-671-3p

PN4427975: RT002322, TM002322

miR-4783-3p

PN4427975: RT463358, TM463358

RNU48

PN4427975: RT001006, TM001006

miRNA-antagmoirs, -mimics, -control reagents – mirVana, Life Technologies

miR-22-3p

#4464084: MH10203

miR-326
#4464066: MC10686
miR-657
#4464084: MH11637
miR-671-3p
#4464084: MH12333
miR-1301-3p
#4464084: MH13788
miR-4704-5p
#4464066: MC22186
miR-4783-3p
#4464066: MC21176
miRNA antagomir negative control
#4464076
miRNA mimic negative control
#4464058

<u>3'UTR luciferase MMP9 oligonucleotide subcloning:</u>
2x wild type-MMP9 sequence
Fwd Primer: gaggCCTGGAGACCTGAGAACCA**A**TCttCCTGGAGACCTGAGAACCA**A**TC
Rev Primer: aaagGA**T**TGGTTCTCAGGTCTCCAGGaaGA**T**TGGTTCTCAGGTCTCCAGG
2x N38S-MMP9 sequence
Fwd Primer: gaggCCTGGAGACCTGAGAACCA**G**TCttCCTGGAGACCTGAGAACCA**G**TC
Rev Primer: aaagGA**C**TGGTTCTCAGGTCTCCAGGaaGA**C**TGGTTCTCAGGTCTCCAGG
2x miR-671-3p Target Sequence
Fwd Primer: gaggGGTGGAGCCCTGAGAACCGGAaaGGTGGAGCCCTGAGAACCGGA
Rev Primer: aaagTCCGGTTCTCAGGGCTCCACCttTCCGGTTCTCAGGGCTCCACC
2x miR-657 Target Sequence
Fwd Primer: gaggCCTAGAGAGGGTGAGAACCTGCCaaCCTAGAGAGGGTGAGAACCTGCC
Rev Primer: aaagGGCAGGTTCTCACCCTCTCTAGGttGGCAGGTTCTCACCCTCTCTAGG
2x scrambled sequence
Fwd Primer: gaggCGCTAGCATAGCTCGACAGACAttCGCTAGCATAGCTCGACAGACA
Rev Primer: aaagTGTCTGTCGAGCTATGCTAGCGaaTGTCTGTCGAGCTATGCTAGCG

Figure S1. Cell culture supernatant loading control validation. **A.** Representative Western blot with graded loading of total cell lysate (μg) or cell culture media (μL) probed with anti-MMP-9 antibody. The total cell lysate membrane was also probed with anti-GAPDH. **B**. Densitometric quantification of total protein input for intracellular and extracellular MMP-9 was linear with GAPDH loading control up to 20 μg of protein loading. Protein loading (total lysate and culture media) of all subsequent experiments were within the linear range of the densitometric analysis.

Figure S2. Expression of wild type- and N38S-MMP9 from the pIRES2-EGFP vector. **A.** Wild type- or N38S-MMP-9 pIRES2-EGFP vectors were transfected into HEK293 cells and phase contrast and fluorescent images captured at 24 hrs right before the cell harvest showed robust EGFP fluorescence. B. The whole cell lysate analyzed by a Western blot probed for MMP-9, EGFP, and GAPDH. The EGFP protein expression was not altered by the MMP-9 inserts but the N38S-MMP-9 protein amount was much reduced. GAPDH served as the loading control.

Figure S3. miRNA-1301-3p and miRNA-22-3p regulation of N38S-MMP-9. **A.** Cartoon illustration of miR-1301-3p and miR-22-3p targeting MMP-9 mRNA. Boxes represent miRNA seed regions; grey highlighted region with "x" represents the location and nucleotide of the N38S polymorphism. **B.** Western blot of extracellular wild type- and N38S-MMP-9 protein in cells treated with 15 or 30 nM miR-1301-3p antagomir, 30 nM scrambled oligonucleotides were added as control. **C.** Western blot of extracellular wild type- or N38S-MMP-9 protein in cells treated with 15 or 30 nM miR-22-3p antagomir, 30 nM scrambled oligonucleotides were added as control. Ant., antagomir; Scram., scrambled.

Figure S4. Antagomir knockdown of endogenous HEK293 miR-671-3p and miR-657. **A.** qRT-PCR of miR-671-3p and miR-657 miRNA levels after 36 hours post-transfection with miR-671-3p antagomir. RNU48 was used as the endogenous control. ****P<0.0001, t-test, n=6. **B.** qRT-PCR of miR-657 and miR-671-3p miRNA levels after 36 hours post-transfection with miR-657 antagomir. RNU48 was used as the endogenous control. ****P<0.0001, t-test, n=5.

Figure S5. Firefly luciferase (fluc) mRNA levels correlate with the enzymatic assay. qRT-PCR of the fluc-miRNA mRNA levels 30 hours post-transfection with the indicated miRNA-luciferase reporter constructs. Firefly luciferase mRNA levels were normalized (denoted as F/R) by the Renilla luciferase mRNA transfection control, n=2.

Figure S6. Detection of miRNA-326- and miRNA-4783-3p-mimic addition to HEK293 cells. **A.** qRT-PCR of miR-326 miRNA levels after 36 hours post-transfection with miR-326-mimic. No Ct detected for scrambled miR-mimic transfection. Values were normalized using 7.5 nM miR-326-mimic to quantify the fold increase. ****P<0.0001, t-test, n=4. **B.** qRT-PCR of miR-4783-3p miRNA levels after 36 hours post-transfection with miR-4783-3p-mimic. No Ct detected for scrambled agomir transfection. Values were normalized using 7.5 nM miR-4783-3p agomir to quantify fold increase. ****P<0.0001, t-test, n=4. RNU48 was used as the endogenous control for both experiments.

Table S1. Oligonucleotides used for MMP-9 studies. MMP-9 cDNA harboring the indicated SNPs were created by either incorporating the polymorphism in a log forward primer (N38S and mS38) or by a standard 2-step PCR protocol (R668Q, G344=) using the common external primers and the indicated internal forward and reverse primers, induced mutations highlighted in red text. The primer pair for qRT-PCR amplified a product of 246 bp common to all constructs. Taqman MicroRNA Assay (Life Technologies) and mirVana (Life Technologies) reagents listed with the product numbers. Tandem MMP-9, miRNA target, or scrambled oligonucleotides were annealed and cloned into the BsmBI site 4mer

overhang within the luciferase 3'UTR. 4mer and linker region highlighted in lowercase text, location of N38S polymorphism or wild type nucleotide indicated with red or green bold text, respectively.

Table S2. SNP-dependent miRNA-targets in Chromosome 20. The Table lists "high stringency" target: miRNA pairs in Chr. 20 selected by ΔScore≥165 or ΔMFE≤-25 kcal/ mol from a less stringent list of target: miRNA pairs identified by the bioinformatics algorithm outlined in Figure 2. For SNPs with 3 polymorphisms, alleles showing the greatest ΔScore and ΔMFE were selected. See **Table 1** legend for explanation of the nomenclature.
